# Insights into Uromodulin and Blood Pressure

**DOI:** 10.1007/s11906-024-01317-0

**Published:** 2024-09-11

**Authors:** Manshi Zhou, Sheon Mary, Christian Delles, Sandosh Padmanabhan, Delyth Graham, Martin W. McBride, Anna F. Dominiczak

**Affiliations:** School of Cardiovascular and Metabolic Health, University of Glasgow, BHF Glasgow Cardiovascular Research Centre, 126 University Place, Glasgow, G12 8TA UK

**Keywords:** Uromodulin, Blood pressure regulation, Hypertension, Mendelian randomisation, Ethnic and sex differences, Biomarker

## Abstract

**Purpose of Review:**

We review the role of uromodulin, a protein exclusively expressed in the kidney, in blood pressure regulation and hypertension.

**Recent Findings:**

The last few years have seen a shift of focus from genetic association to mendelian randomisation and uromodulin-salt interaction studies, thus confirming the causal role of uromodulin in blood pressure regulation and hypertension. This work has been complemented by phenome-wide association studies in a wider range of ethnicities. Important recent molecular work elucidated uromodulin trafficking and secretion and provided more insights into the pathophysiological roles of circulating and urinary uromodulin.

**Summary:**

Uromodulin has a causal role in blood pressure regulation and hypertensin. Recent studies show utility of the uromodulin as a biomarker and a possible precision medicine application based on genetically determined differential responses to loop diuretics.

## Introduction

**Fig. 1:**
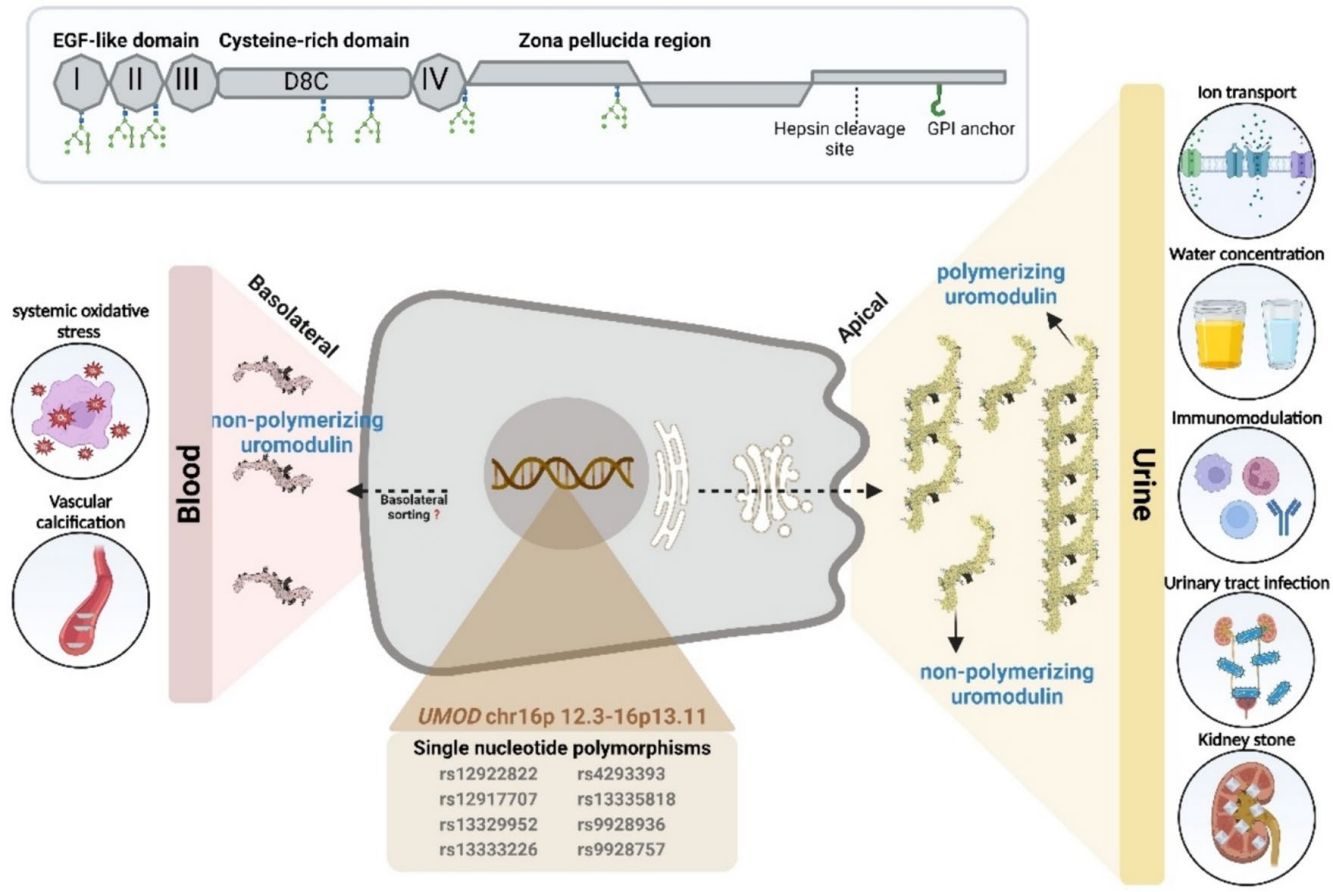
The illustration depicts the production of uromodulin by epithelial cells of thick ascending Loop of Henle (TAL). Human uromodulin, a 640-amino acid protein (top), undergoes extensive glycosylation and is anchored to the cell membrane via a GPI anchor. Following cleavage by the serine protease hepsin at the apical cell surface (right-side), uromodulin is secreted into urine, where it polymerizes to form the matrix of urinary casts. A smaller fraction of uromodulin is also released into the bloodstream through the interstitium, facilitated by the basolateral surface of the cells (left-side). The diverse functions of uromodulin, as depicted by circles in the figure, are complemented by the representation of single nucleotide polymorphisms on the UMOD gene, establishing its connection to hypertension in the illustrated bottom panel

**Fig. 2:**
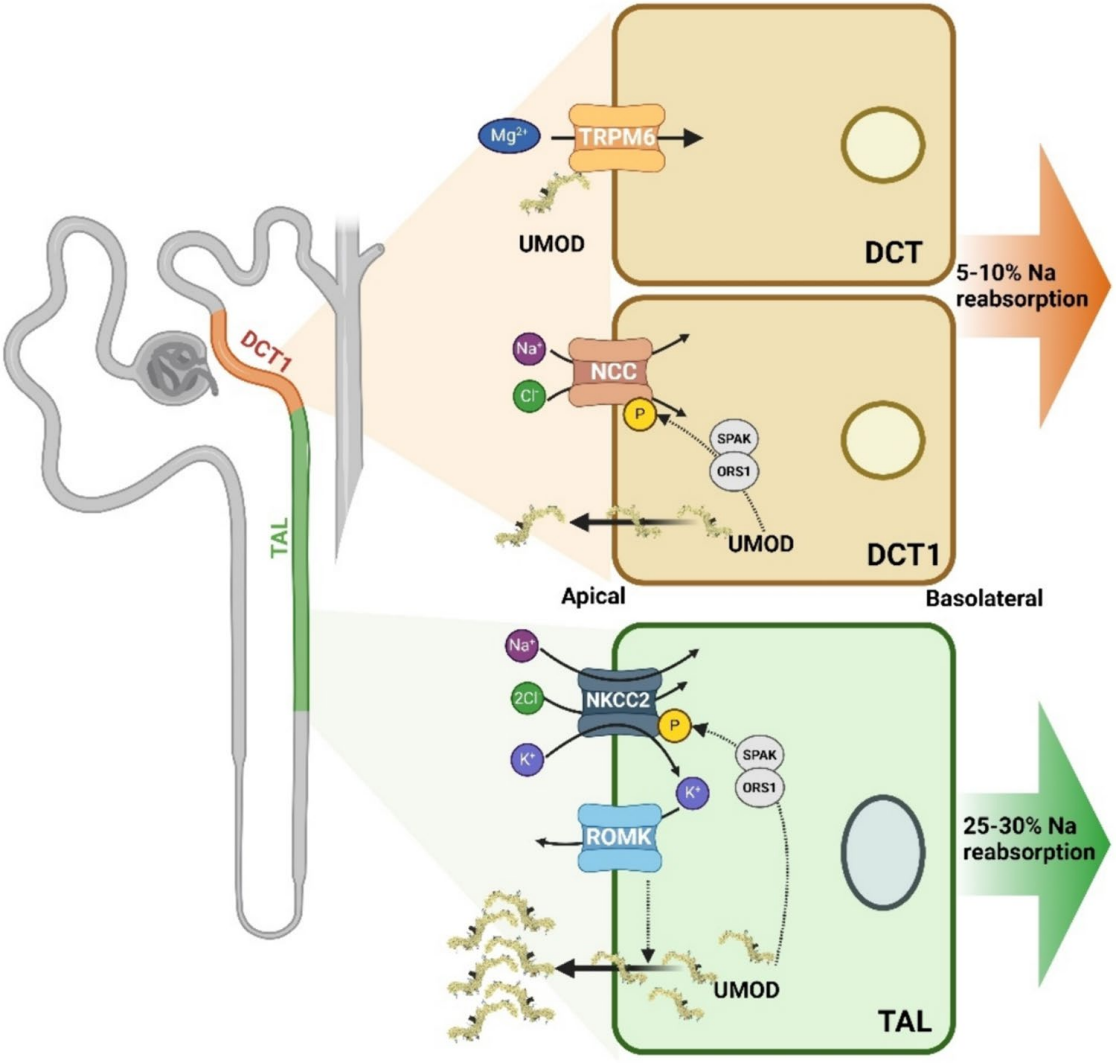
A simplified illustration of the nephron focusing on the thick ascending limb of the loop of Henle (TAL) and the distal convoluted tubule (DCT). UMOD is depicted as being produced in TAL and DCT1 cells and secreted into the urine. Increased UMOD enhances sodium–potassium-chloride cotransporter (NKCC2) activity (via SPAR-OSR1), leading to greater reabsorption of sodium (Na⁺), potassium (K⁺), and chloride (Cl⁻). UMOD enhances sodium-chloride cotransporter (NCC) activity, increasing sodium and chloride reabsorption at DCT1. This results in increased blood volume and pressure, contributing to hypertension. ROMK regulates UMOD apical secretion in TAL. UMOD is also critical for maintaining magnesium homeostasis by regulating TRPM6 activity in the DCT

Uromodulin (UMOD), otherwise known as Tamm-Horsfall protein, is a glycoprotein mostly produced by the thick ascending limb (TAL) and to a lesser extent in the distal convoluted tubule (DCT) [1–3]. The UMOD protein, due to its apical and basolateral bidirectional secretion, plays a role in several biological processes in cardiovascular and kidney diseases (Fig. 1). The majority of uromodulin is secreted apically and aggregates to form polymers in urine [4, 5] whereas some apically secreted and all basolaterally secreted UMOD remain non-polymerized in monomeric or dimeric forms, suggesting different mechanism of trafficking and release into urine, interstitium and circulation (Fig. 2) [3]. One study identified a cleavage at UMOD zona pellucida (ZP) region leading to its inability to polymerize and characterized serum uromodulin (sUMOD) as monomers [6]. Further investigation identified UMOD dimers in both urine and serum, containing the noncleaved external hydrophobic patch (EHP) region suggesting an alternative pathway that is independent of UMOD GPI anchoring and cleavage by hepsin [7]. There have been several excellent recent publications reviewing uromodulin biology and function [8, 9], with a particular focus on chronic kidney disease (CKD) [10]. In this review, we will therefore focus on the relationship between UMOD, blood pressure regulation, and hypertension.

## UMOD, Phenome-wide Association and Mendelian Randomisation

A European population genome-wide association study (GWAS) of blood pressure extremes discovered that the minor G allele of a single nucleotide polymorphism (SNP) (rs13333226) in the cis-promoter of uromodulin was associated with a lower risk of hypertension, lower urinary uromodulin (uUMOD) level and increased estimated glomerular filtration rate (eGFR) [11]. Early series of complementary preclinical animal studies confirmed the involvement of uUMOD in blood pressure regulation and salt-sensitive hypertension. Umod ^− / −^ knock out (KO) mice have significantly lower systolic blood pressure (SBP) compared with 129/sv wild-type (WT) mice on normal chow with little phenotypic changes in the kidney [12]. Crucially, administration of 2% NaCl in drinking water did not alter SBP in Umod ^− / −^ mice, however, SBP increased in WT mice by 33%. In contrast, transgenic mice overexpressing uromodulin exhibit salt-sensitive hypertension and age-dependent renal lesions that are similarly observed in elderly subjects homozygous for UMOD risk variants [13].

Phenome-wide association studies (PheWAS) [14] have been carried out across the UMOD locus using common promoter (rs4293393, rs12917707) and upstream (rs77924615) SNPs searching for associations across 1500 different clinical diagnoses codes mapped to phenotype groups in the Million Veteran Program (MVP) [15]. This very large, multi-ethnic study included 496,961 non-Hispanic Whites (77%), 123,120 non-Hispanic Blacks (19%) and 52,183 Hispanics (8%) with 91% males and 9% females. A key aspect of this study design was the large number of participants, a detailed analysis of frequencies of genetic variants, as well as a replication strategy in two further large studies; the Vanderbilt University Medical Centre DNA Biobank (BioVU) consisting of non-Hispanic White (n = 63,029, 81%) and non-Hispanic Black patients (n = 14,521, 19%), and the proportion of female patients were 56% and 62%, respectively; and the UK Biobank, a prospective cohort study that recruited more than 500,000 volunteers with an average age of 56yrs and 54.4% were female. Hypertension was common in the MVP ranging from 65% and up to 75% and less common in UK Biobank at 27%, although lower levels of event rates in UK Biobank have been addressed and risk factor associations in the UK Biobank are generalisable and compensated by large numbers of participants [16]. In the 1000 genomes project, UMOD promoter and upstream variants from the European population were all within the same linkage disequilibrium block, however, individuals from other African American, Mexican, and Han Chinese populations had different linkage disequilibrium (LD) patterns [17]. In the MVP study, the minor allele frequency (MAFs) of the rs4293393 and rs77924615 variants were 18% and 20% in non-Hispanic White patients, 21% and 6% in non-Hispanic Black patients and 21% and 20% in Hispanic patients, highlighting significant differences in patterns of LD in non-Hispanic Black patients.

In non-Hispanic White participants, the rs4293393 risk variant was associated with significantly increased uromodulin and increased risk of hypertensions (rs4293393; OR: 1.03, 95% CI: 1.05–1.05, P = 2.11 × 10–6) [15]. Other renal and cardiovascular complications of hypertension for UMOD promoter risk variants were also highly significant. Similar findings were observed with other UMOD promoter and upstream variants across this locus and these finding were replicated in the UK biobank, but not the BioVU study. In contrast, UMOD promoter risk variants in non-Hispanic Black patients were not associated with hypertension, renal phenotypes and hypertensive complications. This may be related to differences in patterns of LD at the UMOD locus in different ethnic populations but may also involve processes unrelated to uromodulin. It should also be noted the relatively small number of female participants (9%) in the discovery phase.

Mendelian randomization (MR) is a statistical method used to investigate the causal relationship between an exposure (such as a biomarker) and an outcome (such as a disease) by leveraging genetic variants as instrumental variables [18, 19]. Mendelian randomisation studies have confirmed uromodulin's independent causal role in blood pressure regulation. These studies used genetic variants within with UMOD from GWAS as instrument variables for exposure, and BP and renal function traits from GWAS as the outcomes. Ponte et al. demonstrated that an increase of 1 mg/g in genetically predicted uUMOD/creatinine levels correlates with a decrease of 1 mL/min/1.73 m2 in eGFR, a 6% higher odds of CKD, and modest increases in SBP (0.11 mm Hg) and DBP (0.09 mm Hg) [20, 21]. To quantify uromodulin's independent impact on BP and eGFR, bi-directional and multivariable MR analyses were conducted. These analyses revealed that 28% of the total effect of uromodulin on BP is mediated through eGFR, with the remaining impact being direct [20]. Similarly, You et al. demonstrated that per unit increase of uUMOD and sUMOD contributed to higher odds of hypertension (3.6% and 1.3% respectively) and were causally associated with higher SBP (0.10 and 0.88 mmHg respectively) and higher DBP (0.371 and 0.313 mmHg respectively) [22]. Given the continuous relationship between blood pressure, including throughout the normal blood pressure range, and risk of death from vascular diseases, even small changes in blood pressure can have a dramatic reduction on stroke and ischaemic heart disease mortality [23]. The implication is that even small persistent reductions in blood pressure should prevent large number of premature deaths from vascular diseases. Together, MR studies reported that UMOD variants causally affect SBP, DBP, and eGFR, and a minor proportion of the effect of uUMOD on blood pressure is mediated through eGFR.

It has been suggested that the increase in blood pressure associated with UMOD promoter variants may have a role in cardiovascular disease (CVD). A 2-sample MR study among the general population of European ancestry, aimed to evaluate whether the genetically driven higher uUMOD levels and subsequent increase in blood pressure have a direct causal and positive relationship with CVD risk [24]. The authors identified the effect of higher uUMOD on increasing blood pressure, which mediated a consequent effect on MI risk in the general population, although other CVD phenotypes were not implicated, which may relate to the low statistical power of this study.

Further supporting this, the recent genotype-blinded UMOD trial revealed a potential interaction between UMOD and NKCC2 [25]. In this study with the rs13333226 SNP (AA vs. AG/GG), uncontrolled hypertensive patients with the AA genotype, associated with increased uromodulin levels, showed a significantly greater and more sustained blood pressure response to the loop diuretic torasemide compared to those with AG or GG genotypes [25]. Additionally, AG/GG genotype carriers exhibited more pronounced genotype-specific blood pressure fluctuations, including fall-rebound patterns, over a 16-week period.

## UMOD and Salt Studies

There are several interventional studies looking at salt and uromodulin in humans but methodological differences, sample size, ethnic and sex differences and different study questions impact on the results. The Groningen salt intervention study in young and healthy Caucasian male volunteers (n = 34) reported significantly higher SBP after 7-day high salt diet (200mmol sodium per day) comparing to 7-day low salt diet (50mmol sodium per day) [26]. Higher average uUMOD levels were observed on high salt diet [11]. Comparing UMOD genotypes on a low-salt diet yielded significantly lower uUMOD levels among G (rs13333226) allele carriers compared to homozygous A/A, while high-salt diet showed no differences [11]. This was the first indication of genome-environment interaction. Dietary and urinary sodium are two main measurements required to characterise salt-sensitive hypertension. A uUMOD level dependent association between urinary sodium and blood pressure was demonstrated in Swiss Kidney Project on Genes in Hypertension study [27]. In the highest uUMOD strata (n = 475), higher urinary sodium significantly correlated with higher SBP and DBP. In contrast, the lowest uUMOD strata (n = 473), higher urinary sodium was significantly associated with lower SBP and DBP [27]. The 24-h uUMOD excretion was dependent on rs1297707 genotype but the 24-h urinary sodium was independent [27]. This is important as it provides evidence consistent with UMOD playing a significant role in sodium transport and salt-sensitive blood pressure regulation.

A strict dietary salt intervention study in Chinese individuals (n = 80) reported significantly higher blood pressure and higher urinary sodium after 7-day high-salt diet (307.8 mmol sodium per day) compared to a 7-day low-salt diet (51.3 mmol sodium per day) [28]. However, both sUMOD and uUMOD levels were significantly lower in high-salt intervention than in low-salt intervention when comparing to baseline. A family-based cohort (n = 514) from the same study was used to assess the genetic variants on blood pressure responses. Minor allele of rs4997081 and rs7193058 were associated with DBP and mean arterial pressure in high salt intervention, whereas minor alleles of rs4997081, rs7198000, rs77875418, rs79245268, rs4293393, rs6497476, and rs13333226 were associated with pulse pressure in low salt intervention [28]. Although several UMOD SNPs have been significantly implicated, the study does not consider LD across this locus.

Other salt studies have also been carried out but are difficult to interpret mainly due to relatively small sample size. The DASH-sodium trial reported no differences in uUMOD level among various dietary salt interventions for 30 days in 157 subjects (low-salt: 50 mmol per day; intermediate: 100 mmol per day; high-salt: 150 mmol sodium per day) although no UMOD genotype data was presented [29]. There was a trend to decrease SBP after low salt intervention, but no statistical significance was reported. The larger scale GenSalt study (n = 1906), an earlier salt intervention study on family-based rural Chinese population, incorporated a community dining hall to strictly control daily sodium intake during the study [30]. Younger participants showed smaller increase of SBP than older participants and females showed larger increase than males in the high versus low salt diet [31]. This provides an ideal opportunity for genotyping UMOD promoter variants in this ethnic population; however, such data has not been published thus far.

## Umod Trafficking

In preclinical models, salt is a modulator of UMOD excretion with a decrease in 24-h uUMOD excretion leading to endoplasmic reticulum (ER) retention of UMOD [32]. There was a more significant reduction in uUMOD excretion rate and ER accumulation in the stroke-prone spontaneously hypertensive (SHRSP) rat, a chronic hypertensive model, compared to the contrasting normotensive Wistar Kyoto (WKY) control. This suggests that salt affects UMOD trafficking in both strains, but trafficking is more affected in the SHRSP compared to WKY. Keratin-40 (KRT40), a type I keratin expressed in the kidney also regulates uUMOD trafficking. A meta-GWAS of uUMOD in 29,315 individuals from 13 cohorts of European ancestry reported genome-wide significance at SNP rs8067385 near KRT40 with a minor C allele associated with lower uUMOD [33].

In isolated mouse thick ascending limb (mTAL) segments, both in situ hybridization and immunostaining evidenced the colocalization of KRT40 and UMOD. KRT40 mTAL cell transduction showed the downregulation of KRT40 significantly accumulated UMOD in cell lysates and reduced UMOD secretion in the apical medium. This suggests the expression of KRT40 is positively correlated with uUMOD trafficking. Further insights into UMOD trafficking have been investigated using conditional knock-out animals using non muscle myosin protein NM2A and NM2B encoded by Myh9 and Myh10, respectively [34]. A conditional KO of Myh9 and Myh10 in renal tubules of adult mice resulted in progressive kidney disease. One of the first signs prior to renal tubular damage was the accumulation of UMOD within the tubular epithelium with a loss of apical Na–K-Cl cotransporter (NKCC2) as well as an increase in urinary sodium and potassium, consistent with UMOD affecting localisation of NKCC2 at the apical membrane.

The renal outer medullary potassium channel (ROMK) encoded by *Kcnj1 *showed regulatory effect on uUMOD independent of NKCC2 expression using genetically modified mice and cell culture [35]. *Kcnj1 *KO mouse significantly decreased Umod expression, along with other genes expressed at proximal tubular and TAL, while significantly increasing genes expressed at DCT and the connecting tubule/collecting duct (CNT/CD) [35]. Although significantly lower UMOD protein level was observed in *Kcnj1 *KO urine samples, its level was significantly increased in total kidney lysate confirmed by immunofluorescent labelling. However, NKCC2 expression showed opposite changes in total kidney lysate and immunofluorescent labelling. A decrease of apical UMOD secretion both in ROMK inhibitor treated and in *Kcnj1 *deleted mTAL cells was identified, whereas no changes were present in NKCC2 inhibitor treated samples. Similarly, uUMOD was significantly reduced in Bartter Syndrome Type 2 patients, which harbour inactivating mutations in Kcnj1, with low to normal blood pressure compared to control individuals providing evidence that ROMK directly regulates processing and release of UMOD by TAL cells, independent of NKCC2, relevant for blood pressure regulation [35].

## Serum Uromodulin as a Biomarker

Although sUMOD is recognised in early studies, sUMOD as a biomarker has recently gathered traction as UMOD quantification techniques have improved [8]. Uromodulin variants (rs12917707 and rs12708631) and gene-based analysis were associated with longitudinal blood pressure changes over an eight-year follow up (n = 514) [36]. sUMOD levels were found to be significantly lower in hypertensive participants than in normotensive subjects with a greater reduction in sUMOD as severity of blood pressure increases. This significance remained after adjusting for multiple confounders and irrespective of whether subjects were diagnosed with isolated systolic or systolic/diastolic hypertension. sUMOD was also significantly inversely associated with all-cause mortality, cardiovascular mortality, and myocardial infarction/coronary death in the Cooperative Health Research in the Region of Augsburg (KORA) F4 study on cardiovascular morbidity and mortality (n = 1079) [37]. sUMOD was significantly associated with stroke in males but not females.

The significant associations in males for myocardial infarction/coronary death and females for all-cause mortality and cardiovascular mortality were not maintained after stepwise adjustment. In addition, sUMOD was significantly inversely associated with arterial hypertension with stepwise adjustment for sex, age, eGFR, and cardiovascular risk factors in the KORA F4 study on arterial hypertension (n = 1108) [38]. This inverse association was also observed with the vasoconstrictive prohormone CT-proET-1 with multivariate adjustment, and more pronounced among participants with arterial hypertension compared to normotensive controls. Although sUMOD was inversely related with renin, significance was lost after adjusting for eGFR, cardiovascular risk factors and use of medications.

Participants with higher sUMOD were also less likely to suffer from coronary artery diseases (CAD), heart failure, diabetes, hypertension, or use of anti-hypertensive medication in the Ludwigshafen Risk and Cardiovascular Health (LURIC) study (n = 3057) [39]. Survival analysis adjusted for rs12917707 illustrated higher quartiles of sUMOD associated with lower risk for all-cause motility and cardiovascular mortality. Similarly, cox regression model showed increasing sUMOD concentration strongly lowered risk for all-cause mortality in the total study cohort and in men but not in women. Sex difference was evidenced in patient subgroup analysis, where a more significant association of sUMOD with reduced risk of mortality was found in female compared with male individuals.

Further subgroup analysis identified association of sUMOD with reduced risk of mortality was also shown in patients free from CAD compared with patients with CAD, patients with reduced kidney function compared with patients with an eGFR > 90 ml/min, and patients without diabetes mellitus compared with patients with diabetes mellitus. The risk prediction assessment showed adding sUMOD to established risk prediction scores significantly improved the risk prediction in participants without clinical history of cardiovascular disease [39].

## Concluding Remarks

UMOD research has been reinvigorated by significant association of common promoter and upstream variants in several large-scale genome-wide association studies for hypertension, kidney function and renal disease. Of the numerous loci and genes implicated in the pathophysiology of hypertension, uromodulin has shown promise as a new pharmacological target. This has led to a concerted effort to understand UMOD function in normal physiology and in disease mechanisms using preclinical and clinical strategies.

Considerable progress has been made, but there are many questions still to address. UMOD has been shown to be a multi-functional protein, which has a causal role in the pathophysiology of hypertension. Future studies on the UMOD mechanisms of action and regulation in the circulation and urine will likely yield interesting physiological and pathophysiological outcomes useful in several disease settings, risk assessment and diagnosis. In particular, the role of uromodulin in difficult-to-treat and treatment-resistant hypertension and in advanced cardiovascular conditions that are particularly susceptible to blood pressure such as stroke, ischaemic heart disease and heart failure requires further study.

## Key References

- Micanovic R, LaFavers KA, Patidar KR, Ghabril MS, Doud EH, Mosley AL, et al. The kidney releases a nonpolymerizing form of uromodulin in the urine and circulation that retains the external hydrophobic patch domain. Am J Physiol Renal Physiol. 2022;322(4):F403-f18. This paper identifies the nonpolymerising form of UMOD in urine and circulation that retains the external hydrophobic patch establishing an alternative path for cellular processing and release.
- Schaeffer C, Devuyst O, Rampoldi L. Uromodulin: Roles in Health and Disease. Annu Rev Physiol. 2021;83:477–501. This is an excellent review on UMOD structure and its role in various cardiovascular and kidney diseases.
- McCallum L, Lip S, McConnachie A, Brooksbank K, MacIntyre I, Doney A, Llano A, Aman A, Caparrotta TM, Ingram G, Mackenzie IS, Dominiczak AF, MacDonald TM, Webb DJ, Padmanabhan S. *UMOD* Genotype-Blinded Trial of Ambulatory Blood Pressure Response to Torasemide. Hypertension. 2024 Jul 30. 10.1161/HYPERTENSIONAHA.124.23122. This paper confirm a plausible interaction between UMOD and NKCC2 and suggest a potential role for genotype-guided use of loop diuretics in hypertension management.
- Akwo EA, Chen HC, Liu G, Triozzi JL, Tao R, Yu Z, et al. Phenome-Wide Association Study of UMOD Gene Variants and Differential Associations With Clinical Outcomes Across Populations in the Million Veteran Program a Multiethnic Biobank. Kidney Int Rep. 2022;7(8):1802–18. Robust associations were observed between UMOD promoter and upstream risk variants linked with increased uromodulin expression and increased odds of CKD and hypertension. However, these associations varied significantly across ancestry groups and sex.
- Boehm FJ, Zhou X. Statistical methods for Mendelian randomization in genome-wide association studies: A review. Comput Struct Biotechnol J. 2022;20:2338–51. Very useful review describing the Mendelian Randomisation method.
- Ponte B, Sadler MC, Olinger E, Vollenweider P, Bochud M, Padmanabhan S, et al. Mendelian randomization to assess causality between uromodulin, blood pressure and chronic kidney disease. Kidney Int. 2021;100(6):1282–91. UMOD variants associated with higher levels of urinary uromodulin increase the risk of hypertension and renal disease and support that genetically driven levels of uromodulin have a direct, causal and adverse effect on kidney function outcome and hypertension.
- You R, Chen L, Xu L, Zhang D, Li H, Shi X, et al. High Level of Uromodulin Increases the Risk of Hypertension: A Mendelian Randomization Study. Front Cardiovasc Med. 2021;8:736001. The results indicate that high urinary and serum uromodulin levels are potentially detrimental in elevating blood pressure, and serve as a causal risk factor for hypertension.
- Ponte B, Pruijm M, Ackermann D, Olinger E, Youhanna S, Vogt B, et al. Uromodulin, Salt, and 24-Hour Blood Pressure in the General Population. Clin J Am Soc Nephrol. 2021;16(5):787–9. This study substantiates the role of uromodulin in the salt-sensitive component of BP regulation.
- Du MF, Yao S, Zou T, Mu JJ, Zhang XY, Hu GL, et al. Associations of plasma uromodulin and genetic variants with blood pressure responses to dietary salt interventions. J Clin Hypertens (Greenwich). 2021;23(10):1897–906. This study shows that dietary salt intake affects plasma and urinary uromodulin levels and that uromodulin may play a role in the pathophysiological process of salt sensitivity in Chinese populations.
- Joseph CB, Mariniello M, Yoshifuji A, Schiano G, Lake J, Marten J, et al. Meta-GWAS Reveals Novel Genetic Variants Associated with Urinary Excretion of Uromodulin. J Am Soc Nephrol. 2022;33(3):511–29. This evidenced that lower KRT40 expression led to apical accumulation of UMOD and thus lower uUMOD.
- Otterpohl KL, Busselman BW, Ratnayake I, Hart RG, Hart KR, Evans CM, et al. Conditional Myh9 and Myh10 inactivation in adult mouse renal epithelium results in progressive kidney disease. JCI Insight. 2020;5(21). Conditional non myosin proteins (*Myh9* and *Myh10*) knockouts lead to a UMOD intracellular accumulation and loss of expression of NKCC2 from the apical membrane. Establishes cell type-specific roles for NM2 proteins in regulation of specialized renal epithelial transport pathways.
